# Overview of ACTIV trial-specific lessons learned

**DOI:** 10.1017/cts.2023.698

**Published:** 2024-10-15

**Authors:** Ruxandra Draghia-Akli, Sarah W. Read, Eric A. Hughes

**Affiliations:** 1 The Janssen Pharmaceutical Companies of Johnson & Johnson, Spring House, PA, USA; 2 National Institute of Allergy and Infectious Diseases, National Institutes of Health, Rockville, MD, USA; 3 Teva Pharmaceuticals, Parsippany, NJ, USA

**Keywords:** ACTIV, COVID-19, public-private partnership, master protocols, drug testing

## Abstract

Accelerating COVID-19 Therapeutic Interventions and Vaccines (ACTIV) was an extraordinary example of a public-private partnership (PPP) that brought together over thirty organizations and hundreds of individuals to address one of the most pressing global health needs in recent decades. In particular, ACTIV provided a key avenue for testing numerous therapeutics for their potential benefit in treating the SARS-CoV-2 virus or the resulting symptoms of acute COVID-19 infection. Given the speed and scale at which ACTIV designed and implemented master protocols across global networks that it was simultaneously working to create, the PPP can provide valuable lessons for best practices and avoiding pitfalls the next time the world is faced with a global pandemic of a novel pathogen. This report provides a general overview of the ACTIV partnership to set the stage and context for the subsequent articles in this issue that will relay these lessons learned.

## Key Lessons:

- Engage with all stakeholders early in trial development including funders, pharmaceutical sponsors, implementers, and regulators
- Maintain maximum flexibility in trial design to allow efficient addition of trial arms
- Make focused effort to achieve representative trial populations
- Use pre-established clinical trial networks for efficient study implementation
- Employ extensive outreach and communication plans to publicize trials and ensure timely enrollment

## Back to the future

SARS-CoV-2 infection emerged as a global health threat in 2019 and was declared a pandemic by the World Health Organization (WHO) on March 11, 2020 [1]. The rapid rate of global spread and enhanced mortality led to some of the biggest public health, socioeconomic, and scientific challenges the world had ever faced, well beyond those of previous coronavirus (CoV) outbreaks (e.g., severe acute respiratory syndrome (SARS) in 2003 and Middle East respiratory syndrome (MERS) in 2012).

The response was immediate, and partially informed by the epidemics of the past, such as the Ebola virus outbreaks, including the devastating 2014-2016 outbreak in West Africa [2], and the public health measures undertaken during another pandemic caused by a respiratory pathogen, the 1918 “Spanish” flu [3]. Reflecting on published lessons learned from past outbreaks, it is apparent that the world had again undergone a cycle of “panic and neglect” [4] and was now waking up to the reality that infectious diseases have no borders. Only through national, regional, and global preparation can the risks of pandemics can be attenuated, and their consequences mitigated.

## Partnership, collaboration, leadership – recipe for success

On April 17, 2020, within weeks of the pandemic being declared, the National Institutes of Health (NIH) announced the Accelerating COVID-19 Therapeutic Interventions and Vaccines (ACTIV) public-private partnership (PPP) to develop a coordinated research strategy for prioritizing and speeding development of the most promising treatments and vaccines [5]. Eight US Government agencies, twenty pharmaceutical companies, and four nonprofit partners participated in the PPP (Supplementary Material 1).

**Figure 1.**
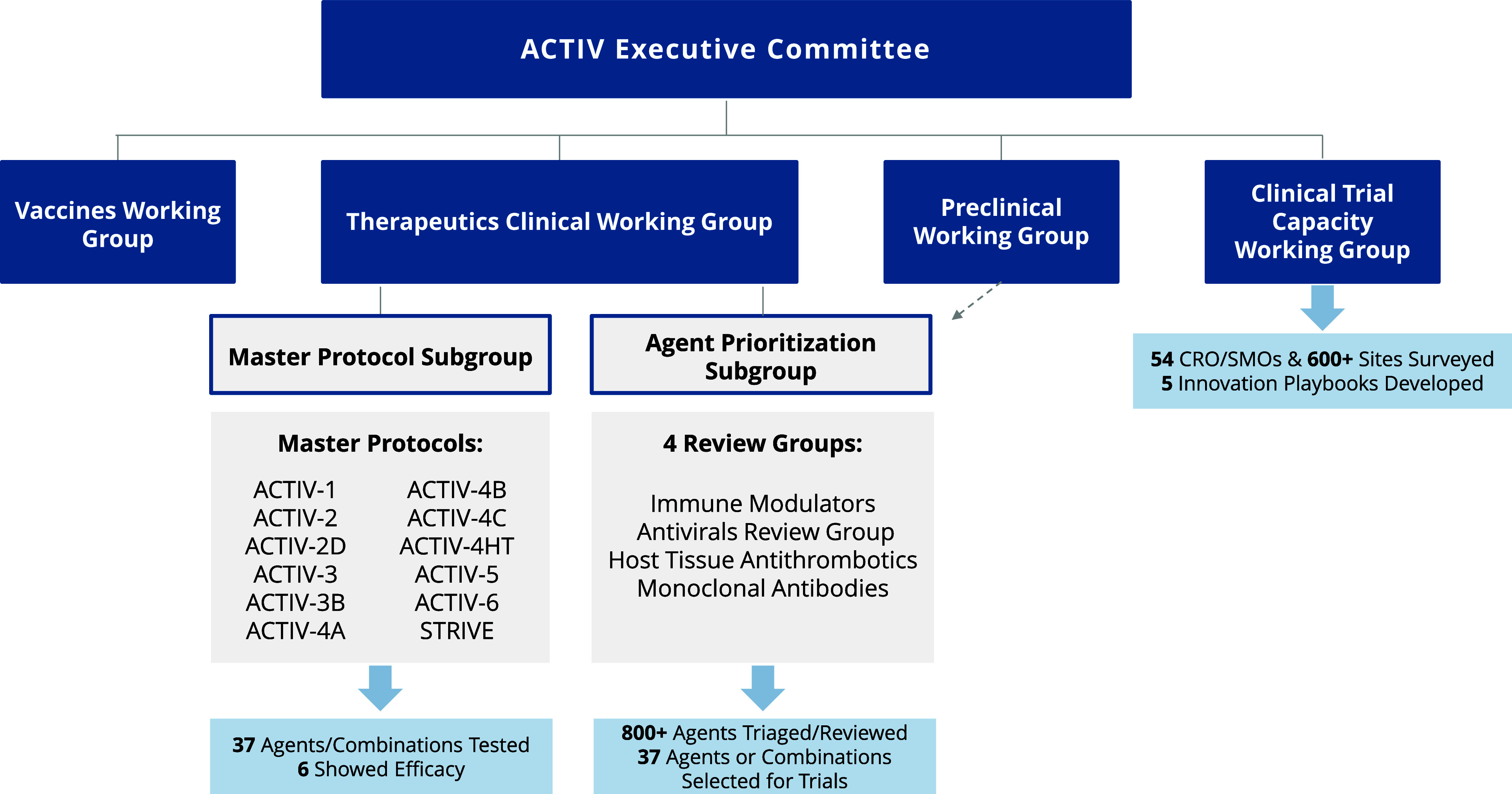
ACTIV organization structure with focus on clinical therapeutics components. Reflected in the figure is an overview of the organizational governance for the ACTIV partnership with a focus on the original working groups that contributed to the launch of the clinical therapeutics testing portfolio, including the Therapeutics-Clinical, Preclinical, and Clinical Trial Capacity Working Groups. The primary outputs of these working groups were review and prioritization of over 800 agents with 37 agents entering the master protocols that were established out of the analysis of numerous NIH and private sector clinical trial networks (54 CRO/SMOs and 600+ sites). While the STRIVE platform is a part of ACTIV, it was set up after the original protocols to continue to respond to COVID-19 post-pandemic and allow for greater pandemic preparedness. ACTIV = Accelerating COVID-19 Therapeutic Interventions and Vaccines; CRO = contract research organization; SMO = site management organization; STRIVE = Strategies and Treatments for Respiratory Infections and Viral Emergencies.

One of the first goals of the ACTIV therapeutic group was to develop a unique and rigorous therapeutic agent intake and assessment process for candidate treatments of acute COVID-19 [6], including antivirals, immune modulators, SARS-CoV-2 neutralizing antibodies, and organ-supportive treatments at both the preclinical and clinical stages of development. As of May 2023, the ACTIV Therapeutics-Clinical Working Group (ACTIV TX-Clin WG) and trial teams have evaluated over 800 therapeutic agents with potential application for coronavirus disease, thirty-eight of which were included for evaluation in one of the eleven protocol platforms that enrolled more than 23,000 patients and was based on a global network of more than 620 sites (Fig. 1). A few agents being studied in the suite of ACTIV trials continue to enroll (with over 26,000 patients) at the time of this publication, but the data cutoff point of May 2023 was timed with the closure of the majority of ACTIV trials.

**Figure 2.**
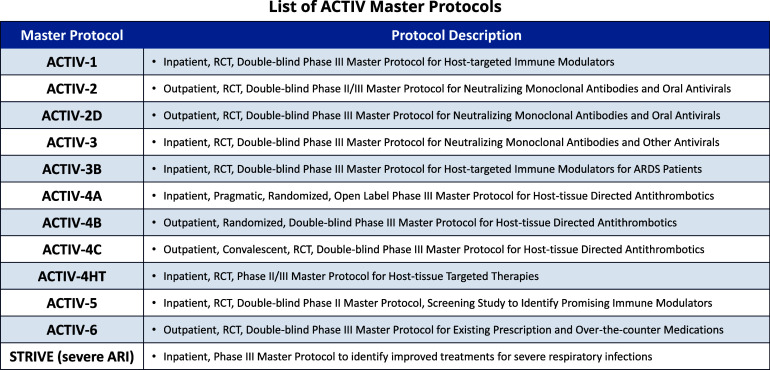
ACTIV therapeutics master protocols. During the COVID-19 pandemic, ACTIV designed and launched the original 11 master protocols that were approved by the FDA. The majority of the trials were double-blind, randomized control trials (RCT) testing novel agents from various patient populations and therapeutic classes. However, the ACTIV-4A protocol used a more pragmatic approach and even collaborated with other global platforms. Despite being a double-blind RCT, ACTIV-6 experimented with a fully-remote, decentralized trial design, which turned out to be quite successful. Finally, all of the lessons learned from the original ACTIV inpatient RCTs were parlayed into the launch of the new STRIVE protocol that is intended to create a global warm-based clinical trial network that can continue to evaluate therapies for COVID-19 patients, as well as create an infrastructure that can pivot to a new response in the event of another pandemic. ACTIV = Accelerating COVID-19 Therapeutic Interventions and Vaccines; ARDS = Acute Respiratory Distress Syndrome; RCT = randomized controlled study; STRIVE = Strategies and Treatments for Respiratory Infections and Viral Emergencies.

In addition to the agent prioritization effort, master protocols were developed to allow for the coordinated and efficient evaluation of multiple prioritized therapeutic agents. The protocols were designed using a portfolio approach to serve the following patient populations with COVID-19: mild to moderately ill outpatients, moderately ill inpatients, and critically ill inpatients [7] (Fig. 2).

ACTIV’s efforts were complemented by those undertaken by the WHO which developed the Solidarity platform trial [8], the UK government which developed the Recovery Trial [9], and additional platform trials including the Adaptive COVID-19 Treatment Trials [10], Inpatient Treatment of COVID-19 With Anti-Coronavirus Immunoglobulin [11], Convalescent Plasma in Outpatients With COVID-19 (C3PO) [12], CONTAIN COVID-19 [13], ANTICOV [14], TOGETHER [15], Investigation of Serial studies to Predict Your Therapeutic Response with Imaging And moLecular Analysis (I-SPY COVID) [16], and Randomized, Embedded, Multifactorial Adaptive Platform Trial for Community-Acquired Pneumonia (REMAP-CAP) [17].

Together, our collective knowledge about the pathophysiology of the disease, patient populations, treatment options, clinical trial protocol design, and implementation has evolved significantly in the last few years. Of note, some trials yielded positive results, and those interventions were immediately implemented in guidelines and clinical practice, saving lives (e.g., thromboprophylactic low-molecular-weight heparin [18] and dexamethasone [19]). Equally important, or even more so, negative results helped winnow the field of candidate therapeutics of those that do not help, and could potentially harm patients with COVID-19, both directly and indirectly, by denying them genuine treatments [20], while also combating misinformation.

It should be noted with the evolution of SARS-CoV-2 variants, monoclonal antibodies (mAbs) which proved useful early in the pandemic, became less so as the virus mutated and evolved, causing a need for continuous innovation in the space and highlighting the need to develop multiple treatment options, such as small molecules and host-directed therapies.

**Figure 3.**
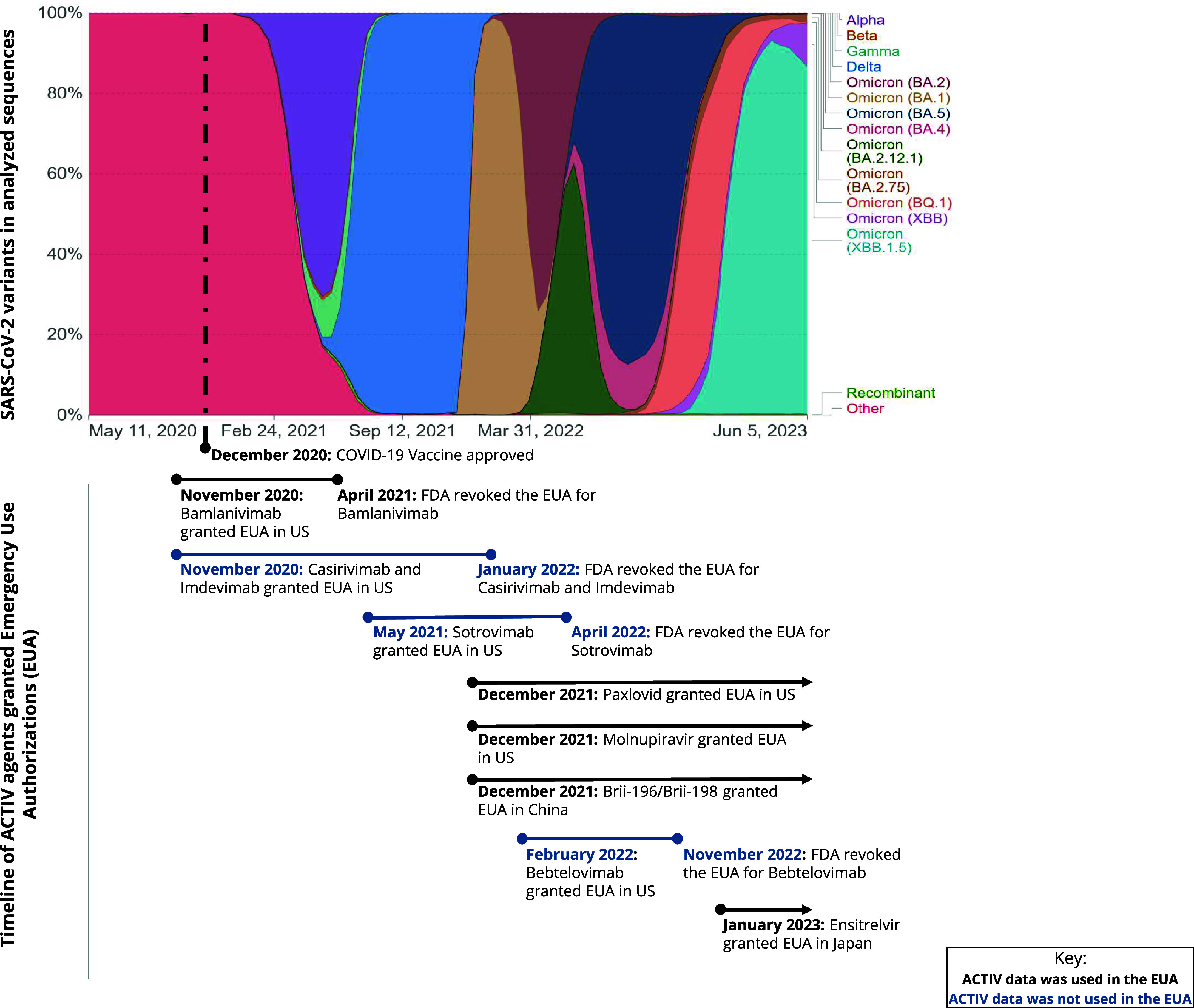
Timeline of the rise and fall of variants and approval of when different agents within ACTIV were granted EUAs. Permissions received to use the variant chart sourced from Our World in Data. SARS-CoV-2 sequences by variant, Sep 11, 2023 (ourworldindata.org). Data from the ACTIV master protocols contributed to some of the key EUAs during the pandemic both in the US and other countries. However, with the evolution of the pandemic, the efficacy of some of these agents waned supporting the need for standing platforms and trials to test novel agents against SARS-CoV-2 and its resulting disease pathobiology. ACTIV = Accelerating COVID-19 Therapeutic Interventions and Vaccines; EUA = Emergency Use Authorization; FDA = Food and Drug Administration; US = United States of America.

The evolution of the virus and emergence of new variants required continuous tracking of therapeutic activity as additional variants arose, and the NIH launched the Tracking Resistance and Coronavirus Evolution project to do just that. The initiative surveyed new viral variants, assessed vaccine and therapeutic resistance, and evaluated the impact of genetic variation on viral biology and on the clinical approaches for preventing and treating illness [21] (Fig. 3).

Furthermore, when it became apparent that some patients experienced debilitating long-term symptoms after a SARS-CoV-2 infection, Researching COVID to Enhance Recovery (RECOVER) was launched. The goal of RECOVER is to rapidly improve our understanding of and ability to predict, treat, and prevent post-acute sequelae of SARS-CoV-2 (PASC), including long COVID [22].

## Lessons learned

The collection of papers in this journal supplement summarizes the lessons learned regarding multiple facets of the design and implementation of master protocols by the ACTIV TX-Clin WG and ACTIV trial teams and provides a set of recommendations for areas deemed essential for pandemic preparedness. The hope is that the world will act on these lessons, as well as those learned by other groups who participated in the COVID-19 research response, in order to better prepare for the next pandemic and avoid yet another cycle of panic and neglect. As a global community, we need sustained funding for all aspects of preparedness and response, from research to manufacturing; clinical trials infrastructure that is kept “warm” in the United States and around the globe; and end-to-end, discovery-to-implementation, results-driven public-private partnerships.

## Importance of appropriate representation in enrolled populations

**Figure 4.**
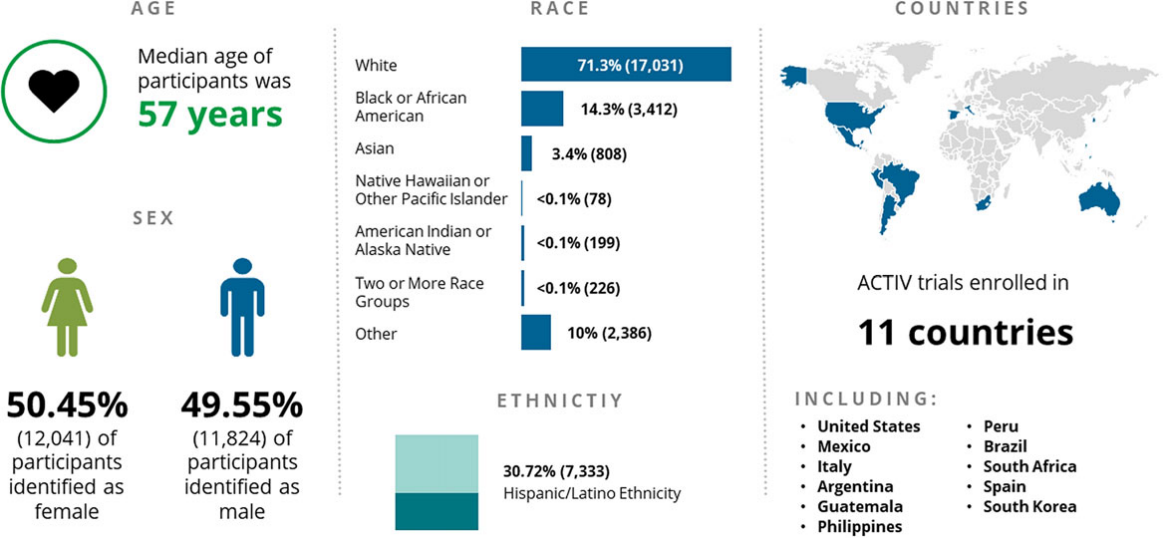
ACTIV trial summary demographics as of May 2023. Several trials have continued to enroll since May of 2023, so the total ACTIV trial enrollment is now over 26,000 participants from 11 countries across the globe with 3 of the original ACTIV master protocols still in operation as of this report submission (ACTIV-2D, ACTIV-4HT, and ACTIV-6). As shown by the data, across the ACTIV trials the median age of the trial participants was 57 years old, in line with the fact that SARS-CoV-2 more greatly effected older populations. The split between enrollment of male and female participants was virtually equal, and the predominant race that was recruited was White/Caucasian (71.3%). ACTIV had reasonably strong recruitment of Black/African American participants across the trials (14.3%) but had smaller populations of other races. Improvement in the diversity of race recruitment will be a strong target for future efforts. One area where ACTIV saw strong diversity in recruitment was among the Hispanic/Latino ethnicity (30.72%). The tactics for this success are discussed in the Outreach, Recruitment, and Engagement manuscript. ACTIV = Accelerating COVID-19 Therapeutic Interventions and Vaccines.

During the course of the pandemic, knowledge of the pathophysiology of the disease evolved, as did available treatments. The burden of COVID-19 was largely experienced by older individuals, and people of color, particularly those in underserved communities, both in the United States and across the globe, with hospitalizations and deaths significantly higher among these groups [23]. Although some of the ACTIV trials enrolled diverse populations, which contributed to the better understanding of the disease, others were less successful. Many of the lessons learned described in this supplement are focused on how to better achieve representative patient populations within the enrolled trial participants. Questions remain regarding how to better implement the lessons learned and translate them into actual benefits for the concerned populations (Fig. 4).

The opening of clinical trial sites in Latin America resulted in increased inclusion of Hispanic participants, while other populations, such as Native Americans, or those of mixed backgrounds were less represented. Despite sustained efforts, the outpatient trials tended to enroll predominantly White people.

Some questions are still outstanding: did we include large enough numbers from minority groups to allow sufficiently powered subgroup analyses? Are some patient groups under-represented because they are less likely to agree to participate in clinical trials? Even without the answers to these questions, we can document which strategies were effective in increasing diversity in trial populations.

## Considerations for protocol design and implementation

Contributions to this special supplement include reflections on lessons learned regarding protocol design and implementation. Therein, the authors lay out strategies for approaching the multiple critical decisions to be made when designing master protocols to test therapeutic candidates in the setting of a pandemic, particularly when the pandemic is caused by a novel pathogen and knowledge of the pathogenesis and clinical course are rapidly evolving.

One of the important lessons learned regarding trial design is to maintain maximum flexibility allowing for greater efficiency when implementing different study arms as the pandemic evolves. Some examples include allowing for nonrestrictive eligibility criteria, collecting data related to multiple endpoints, and allowing for a broadly defined standard of care. In addition, forestalling finalization of an analysis plan as long as possible allows for the most relevant endpoints and analysis approaches at the point at which a trial is completing.

Early engagement with stakeholders is of great importance and again allows for efficiency. Active engagement between the public funders, coordinators of the trials, and pharmaceutical sponsors assures that critical information about investigational products is considered in the design, and communication with community partners helps to ensure acceptability of the trial. Finally, early participation of a multi-disciplinary team allows for consideration of all practical aspects of trial design.

The implementation of master protocols comes with challenges. Foreseeing and planning around these challenges can aid in faster study startup and expeditious completion of studies. Use of pre-established networks of clinical trial sites is likely the most important factor toward efficient protocol implementation, and measures such as ongoing funding to keep networks active are critical for pandemic preparedness. Additional measures related to the clinical trial sites such as establishment of expected timelines, use of checklists for standard operations, rapid provision of funding, and flexibility in funding-related contract terms are to be considered. Development ahead of time of template contracts could significantly cut down the time to activate networks and clinical trial sites.

Frequent, regular communication with sites as well as with relevant communities will aid in protocol implementation and recruitment. A channel for having questions answered quickly and collecting feedback in real-time is critical. Consideration should be given toward standardized training for staff and education for affected communities.

Communication with relevant regulatory bodies throughout the protocol design period is another critical lesson learned. Having early and frequent feedback from regulatory partners helps in designing trials from which the data will be actionable. Ideally, such communication channels should be established pre-pandemic to establish mutually acceptable design considerations to rapidly employ when the next pandemic strikes.

Engagement and communication with affected communities were key to increasing public awareness of the ACTIV trials as well as timely completion of enrollment. Use of multiple engagement strategies by protocol teams proved necessary including use of websites, call centers, digital search engine marketing, and display ads. Input from Community Advisory Boards and Stakeholder Advisory Committees comprised of members of affected communities as well as care providers assured the appropriateness and effectiveness of outreach strategies.

## Applying lessons toward preparedness

The ACTIV PPP ultimately implemented eleven master protocols each evaluating multiple candidate therapeutics. Each trial was designed to meet a unique clinical research need but many experienced common challenges. The many ACTIV members, investigators, and study teams who invested thousands of hours in designing and implementing these trials have now reflected upon their experience and summarized their recommendations and strategies for how to improve the research response when the next pandemic strikes. The ACTIV PPP has indeed begun to act on its own lessons learned regarding maintaining clinical trial infrastructure by creating the ACTIV Strategies & Treatments for Respiratory Infections & Viral Emergencies (STRIVE) protocol [24], which was designed to identify improved treatments for severe respiratory infections including antivirals, passive immunity agents, immunomodulators, host-directed therapies, combination therapies, repurposed drugs, novel drugs and supportive care approaches, and which can be employed in the case of a newly emergent pandemic. It is the hope of the ACTIV PPP that all relevant stakeholders will act now to take advantage of these learnings and turn them into a new approach to learning and applying lessons for preparedness.

## Supporting information

Draghia-Akli et al. supplementary material Draghia-Akli et al. supplementary material

## Supplementary material

The supplementary material for this article can be found at https://doi.org/10.1017/cts.2023.698.
