# Supplementary material for: Overview of ACTIV trial-specific lessons learned
Source: J Clin Transl Sci. 2024 Oct 15;8(1):e149. doi: 10.1017/cts.2023.698 (PMC11523021; doi:10.1017/cts.2023.698)
Supplement: Draghia-Akli et al. supplementary material [file S2059866123006982sup001.pdf]

ACTIV Private-Public Partnership Stakeholders

8
Government Partners

- Biomedical Advanced Research and Development Authority
- Centers for Disease Control and Prevention
- European Medicines Agency
- National Institutes of Health
- U.S. Army Medical Research and Development Command
- U.S. Department of Veterans Affairs
- U.S. Food and Drug Administration
- White House COVID-19 Response Team

39
Industry Partners

Original ACTIV Partners

- AbbVie Inc.
- Amgen
- AstraZeneca PLC.
- Bristol-Myers Squibb Co.
- Eisai Co.
- Eli Lilly and Co.
- Evotec BioSystems GmbH
- F. Hoffmann-La Roche AG
- Gilead Sciences, Inc.
- GlaxoSmithKline
- Johnson & Johnson, Inc.
- Merck & Co., Inc.
- Moderna, Inc.
- Novartis AG
- Novavax, Inc.
- Pfizer, Inc.
- Rhythm Pharmaceuticals, Inc.
- Sanofi S.A.
- Takeda Pharmaceutical Industries, Ltd.
- Vir Biotechnology, Inc.

Additional Contributing Partners

- Accord Healthcare
- Alexian Pharmaceuticals
- Apotex
- Brii Biosciences
- Constant Therapeutics
- Deloitte
- Humanigen, Inc.
- Ingenus Pharmaceutical
- Molecular Partners
- NeuroRx
- Rhthym Pharmaceutics, Inc.
- Rigel Pharmaceuticals, Inc. (RIGL)
- Rose Li Associates
- SAB Biotherapeutics
- Sagent
- Shionogi, Inc.
- Synairgen
- Teva Pharmaceuticals
- Trevena, Inc.

4
Non-Profits

- Bill & Melinda Gates Foundation
- Foundation for the National Institutes of Health
- Fred Hutchinson Cancer Center
- Research Triangle Institute

Supplementary Material 1: ACTIV Private-Public Partnership Stakeholders. The success of ACTIV relied on the countless hours and contributions from the valued members of the PPP. As a trusted partner and coordinator of ACTIV, FNIH was able to bring critical government, industry, and non-profit organizations together to work towards creating a framework of clinical networks and designing and implementing the ACTIV Master Protocols.

ACTIV = Accelerating COVID-19 Therapeutic Interventions and Vaccines, PPP = Public-Private Partnership, US = United States of America.
